# Recent Progress in Modification Strategies of Nanocellulose-Based Aerogels for Oil Absorption Application

**DOI:** 10.3390/polym14050849

**Published:** 2022-02-22

**Authors:** M. A. Iskandar, Esam Bashir Yahya, H. P. S. Abdul Khalil, A. A. Rahman, M. A. Ismail

**Affiliations:** 1 School of Physics, Universiti Sains Malaysia, Penang 11800, Malaysia; iskandar@greentagro.com (M.A.I.); arazhar@usm.my (A.A.R.); 2 School of Industrial Technology, Universiti Sains Malaysia, Penang 11800, Malaysia; essam912013@gmail.com; 3 Teraju Saga Sdn. Bhd. MP813, Jalan Melaka Perdana 2, Taman Melaka Perdana, Alor Gajah, Melaka 78000, Malaysia; ikrammki00@gmail.com

**Keywords:** nanocellulose aerogels, modification techniques, environmental wastes, oil absorption

## Abstract

Oil spills and oily wastewater have become a major environmental problem in recent years, directly impacting the environment and biodiversity. Several techniques have been developed to solve this problem, including biological degradation, chemicals, controlled burning, physical absorption and membrane separation. Recently, biopolymeric aerogels have been proposed as a green solution for this problem, and they possess superior selective oil absorption capacity compared with other approaches. Several modification strategies have been applied to nanocellulose-based aerogel to enhance its poor hydrophobicity, increase its oil absorption capacity, improve its selectivity of oils and make it a compressible and elastic magnetically responsive aerogel, which will ease its recovery after use. This review presents an introduction to nanocellulose-based aerogel and its fabrication approaches. Different applications of nanocellulose aerogel in environmental, medical and industrial fields are presented. Different strategies for the modification of nanocellulose-based aerogel are critically discussed in this review, presenting the most recent works in terms of enhancing the aerogel performance in oil absorption in addition to the potential of these materials in near future.

## 1. Introduction

Water pollution from various organic pollutants including frequent oil spills and other synthetic chemical leaks has recently caused significant environmental issues, which become worse every year due to the accumulation of such pollutants in the environment [1]. Conventional approaches that depend on synthetic and petroleum-based polymers, which are nonbiodegradable materials, could introduce another source of environmental pollution [2]. These concerns have encouraged scientists and catalyzed research into green alternatives with improved property profiles. Oily wastewater, as one of the most challenging environmental issues that require many treatment efforts, can be caused by either industrial or domestic factors [3]. Crude oil production is the main cause of industrial oily wastewater, in addition to oil refineries, compressor condensates, petrochemical industry, car washing, metal processing and the use of lubricants and cooling agents in other industries [4]. On the other hand, domestic oil and grease mainly come from total organic matter [5]. In this context, many approaches have been developed for oily water treatment and oil/water separation, including biological degradation by using specific microorganisms to degrade pollutants dissolved in effluents [6]. However, the efficiency of microorganisms in oil degradation is very limited compared with that in the degradation of other pollutants. Other researchers suggested in situ burning of oils; however, this method cannot remove the majority of oil, and it will cause environmental pollution [7]. Currently, physical absorption and membrane separation are the most used methods for oil/water separations and have attracted the greatest attention considering the drawbacks of the other approaches as they are effective and reliable with high absorption efficiency and little adverse environmental effect [8,9,10]. Thus, researchers nowadays are working on developing ecofriendly and cost-effective absorbents that selectively absorb oil with good reusability without significant reduction in absorption capacity.

**Figure 1:**
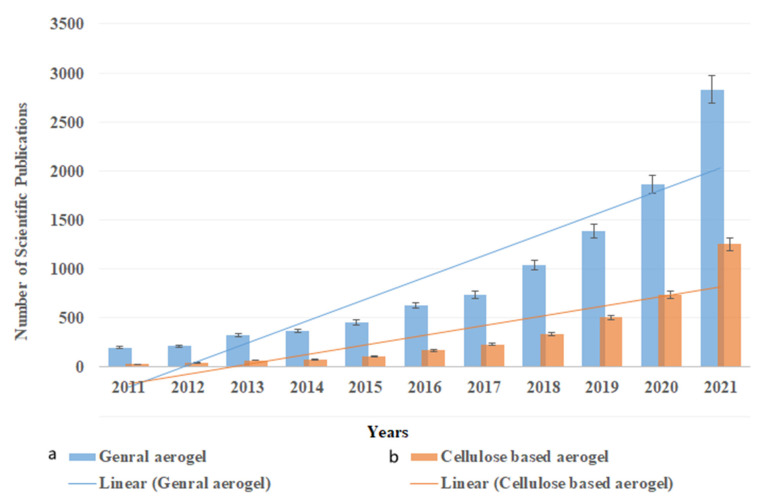
Number of scientific publications in the past 10 years on the use of aerogels as absorbent materials: (**a**) general aerogel; (**b**) cellulose-based aerogel. The research was done using the ScienceDirect database on 30 December 2021, using the keywords (Aerogel absorption) for general aerogel and “cellulose aerogel absorption” for cellulose-based aerogels.

Aerogels are highly porous functional materials that have been prepared from organic and inorganic materials and applied for numerous applications, including water treatment. Nanocellulose-based aerogel is known for its high porosity, ultralow density and excellent absorption capacity [11]. As a biopolymer, cellulose is a biodegradable, cost-effective and ecofriendly substance, and developing nanocellulose aerogel only consumes a tiny amount of cellulose due to the high porosity of aerogels [12]. Thus, the past few years witnessed a significant increase in the studies that utilize nanocellulose as precursor material in aerogel fabrication for different absorption applications. Figure 1 presents the number of scientific publications in the past 10 years on the use of aerogel as an absorbent functional material and the utilization of cellulose in this field, which are expected to highly increase in the next five years. Although several other biopolymers have also been used in aerogel fabrication and as absorbent materials, cellulose retains the attention of scientists due to its unique properties.

Despite the extraordinary advantages of nanocellulose and aerogels, pure nanocellulose typically suffers from very poor hydrophobicity in addition to inferior mechanical properties [13]. Nanocellulose aerogels are not suitable in their pure form for any water treatment application, due to their high hydrophilicity and poor mechanical stability underwater. Therefore, great effort has been made in the modification of nanocellulose to suit water treatment applications and to even enhance its selective absorption and separation performance. Scientists are convinced that nanocellulose aerogels will play a significant role in the near future in terms of oil absorption and separation. Most of the current studies are only conducted in the lab to develop novel functional materials and investigate their performance compared with conventional ones. Several approaches were developed to change the nature of nanocellulose aerogel to become superhydrophobic, enhance its compressibility and elasticity and make it magnetically responsive for easier recovery and reusability. Several review articles have been published regarding the synthesis and characterization of highly hydrophobic, oil-absorbing aerogels [14] and aerogels for general application and water treatment applications [15]. Zamparas et al. [16] discussed the modification methods for improving the hydrophobicity of natural sorbents without specifying any type of biopolymers. Zaman et al. [17] covered the preparation, properties and general applications of cellulose-based aerogels without focusing on the oil separation. This review presents an overall introduction to nanocellulose-based aerogels, their fabrication and their different applications and covers in detail the most recent techniques for the modification of nanocellulose-based aerogels to suit oil absorption applications. It presents the most recent works in this field and discusses the potential and prospective uses of such materials in the near future.

## 2. Nanocellulose-Based Aerogels: Properties and Application

The term “nanocellulose” encompasses three major cellulose-based materials, namely cellulose nanofibers, cellulose nanocrystals and cellulose nanoparticles, which all can be isolated by various isolation techniques [18]. The isolation technique has been reported to affect the properties of nanocellulose; using chemical, mechanical and/or enzymatic treatments of the precursor material could affect its surface functional groups, thermal stability and mechanical properties. Apart from the isolation technique, the properties of nanocellulose were also found to depend on the source of precursor material and potential subsequent surface transformations [19]. Many researchers nowadays use surface modification techniques for nanocellulose to enhance its properties in different materials, including aerogels, by introducing charged or hydrophobic moieties [20]. Since the properties of aerogel are mainly determined by the source of precursor material, choosing the appropriate material is important to achieve a high-performance functional material.

### 2.1. Properties of Nanocellulose-Based Aerogels

As the most abundant biopolymer on earth, cellulose has received the attention of scientists in different fields, including biodegradable plastics, optical films, modern coatings, composites and laminates and controlled release of actives, in addition to membranes and related separation media [2,21]. It has been utilized in the fabrication of different functional materials such as films, membranes, coating agents, hydrogels and aerogels [22]. Many researchers have categorized nanocellulose materials into three main types, namely nanofibrillated cellulose, nanocrystals and nanoparticles, in addition to bacterial cellulose, which is another type of cellulose produced by certain bacteria such as *Acetobacter xylinum* as an extracellular product. Bacterial cellulose is very pure cellulose with narrow size distribution and high crystallinity [23,24]. All the cellulose types have relatively similar chemical compositions but different degrees of crystallinity, particle sizes and morphological properties [25]. Pure nanocellulose aerogel possesses closely similar properties to dried cellulose fibers depending on the effect of fabrication technique on the nanocellulose [26]. As a three-dimensional (3D) fiber network, aerogel is mainly composed of the precursor material(s) after the solvent is removed from the system. Nanocellulose is known for its unique physical, chemical and thermal properties, in addition to its availability, ecofriendliness and nontoxicity. In a recent study, Rizal et al. [26] reported that pure nanocellulose aerogel possessed such a high water absorption ability that it could not remain intact underwater. The authors also added that introducing chitosan into the nanocellulose aerogel enhanced its underwater stability and made the aerogel remain intact underwater. Similarly, Kontturi et al. [27] reported that nanocellulose and its materials are characterized by hydrophilicity and underwater superoleophobicity due to the unique face chemistry and crystallinity of nanocellulose. However, the properties of any aerogel are said to be determined by the precursor materials and their origin, the fabrication approach and the additives [28,29]. The porosity and mechanical strength of pure nanocellulose aerogel can be adjusted by changing the initial concentration of nanocellulose [30]. In a recent study, different concentrations of nanocellulose were used in aerogel preparation using vacuum infusion with biobased epoxy [31]. The study revealed that initial nanocellulose concentration and density were directly proportional with the surface area and porosity were inversely proportional.

Generally, oil/water separation materials based on special wettability are divided into two kinds: hydrophobic oleophilic and hydrophilic oleophobic materials [32]. Hydrophobic oleophilic materials characterized by the rapid spread of oil droplets upon an oil/water mixture touching the surface of that material. Water does not wet the surface of hydrophobic oleophilic materials and is intercepted on the impermeable membrane, in the so-called oil-removing method [33]. However, hydrophilic oleophobic materials are the opposite, as they form a water isolation layer on their surfaces. Nanocellulose must be cross-linked with another material to modify the hydrophilicity of nanocellulose and to improve its mechanical stability. Other researchers have incorporated different materials such as titanium dioxide (TiO₂) [34], nano-silica [35], hydroxyapatite [36] and graphene [37] into nanocellulose to enhance its properties in water treatment applications. Tang et al. [38] recently modified pure cellulose aerogel by directly mixing the raw nanocellulose with poly(propylene glycol adipate), which is a polyester plasticizer. The authors were able to enhance the mechanical strength of the aerogel in addition to its adsorption capacity for dyes. The property enhancement of nanocellulose aerogel continues to satisfy sophisticated requirements for different water treatment applications [39]. Loading of nanocellulose aerogel with a functional material that could enhance both the mechanical properties and the adsorption capacity at the same time is attracting great attention for its potential in easing the fabrication and minimizing the cost of production. The morphology and pore size of nanocellulose aerogels are two important characteristics that typically influence the absorption capacity of the material; they are determined by several factors, including precursor material(s), preparation approach, additives and/or modifiers, cooling rate and physical conditions such as drying [40]. In a recent study by Sakai et al. [41], the authors reported that supercritical drying of nanocellulose aerogel led to the generation of smaller and open pores compared with other drying approaches such as freeze-drying. Nanocellulose-based aerogels have been also utilized in medical applications due to their biocompatibility, noncytotoxicity, nonimmunogenicity and biodegradability; the past few years witnessed the use of such aerogels in drug delivery, tissue engineering, biosensing and wound dressing applications [42,43,44]. Nanocellulose-derived carbon aerogels are another form of aerogels that exhibit high adsorption capacity and are usually prepared by pyrolyzing the precursor materials [45]. Spongelike carbon aerogel from nanocellulose, with high porosity (99%), ultralow density (0.01 g/cm^3^), hydrophobic properties (149° static contact angle) and reusability, has been reported by Meng et al. [46]. The authors reported that the carbonization process removed hydrophilic functional groups from the cellulose, making the resulting aerogel highly hydrophobic with extraordinary ability to absorb oil.

### 2.2. Fabrication of Nanocellulose-Based Aerogels

**Figure 2:**
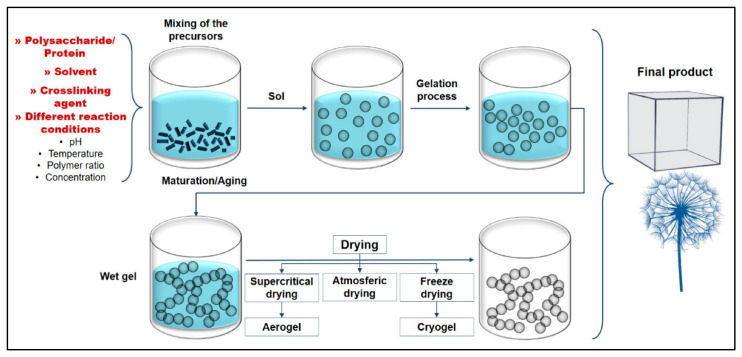
Illustration of the conventional fabrication process of aerogels: dissolving and mixing of biopolymer(s), hydrogel formation, aging and drying process. Adapted from [49], with permission from MDPI, 2022.

As a 3D structured material, the main factor of fabricating aerogels with high porosity and desired stability is to remove the solvent from the polymeric network without any structural disorder. To achieve this, various fabrication techniques have been developed (extensively reviewed by Abdul Khalil et al. [47]), which all initially were established on the formation of polymeric gel and the drying of that gel using supercritical conditions. Supercritical conditions allow the solid form to directly transfer to gas without passing through the liquid state, ensuring the shape of the precursor network structure remains. Depending on the precursor material(s), this process consists of several steps, which can be summarized as dissolving the precursor polymer(s) in water or any suitable solvent, followed by gelation, aging and finally drying (Figure 2) [48]. It has been reported that each step is highly affected by related parameters; chitosan does not dissolve in distilled water and requires weak acid, compared with carrageenan and agar. Pure cellulose does not form gel without cross-linkers, and thus for pure cellulose aerogel, immediate freezing of a homogeneous suspension is required before the freeze-drying process [40].

Various recent investigations found that type of precursor, its concentration, the type and concentration of solvent(s), pH, temperature and the drying method play a key role in tuning the characteristics and properties of the prepared aerogel structure [48,50]. Among all these parameters, selecting a suitable drying approach is the most important parameter determining the aerogel characteristics. High-temperature and low-temperature supercritical drying are the most used techniques for drying polymeric gels, in addition to freeze-drying and ambient-conditioned evaporation. Although using supercritical carbon dioxide drying for the fabrication of nanocellulose aerogel was effective, it has the drawbacks of being dangerous (due to the use of high pressure), time-consuming and relatively expensive [51]. The sol–gel route of aerogel fabrication was developed in the 1960s by replacing water glass with tetramethoxysilane (TMOS), which makes the removal of supercritical fluid from the gel system easier. Pure nanocellulose does not form thick gel without the use of a chemical cross-linker; thus, the freeze-drying technique is highly suitable for its preparation. Upon freezing the nanocellulose suspension, this approach removes water and ice crystals without the need for high temperature or any other organic solvents. The porosity, pore size and pore shapes can be controlled by changing the concentration of the nanocellulose material. Venkatesan et al. [52] reported that energy consumption can be a drawback associated with the freeze-drying technique, in addition to irregular pore size and shape. In a different study, Kanimozhi et al. [53] compared this technique with salt leaching in the nanocellulose composite aerogel fabrication and revealed a better control of pores by using the salt leaching technique. Such conventional techniques for aerogel preparation require the use of organic solvents and salts, may affect the properties of the material and could lead to chemical pollution. The past two decades witnessed great advances in aerogel preparation techniques using advances in technology aided by computers, known as rapid prototyping techniques. Selective laser sintering, stereolithography, injection molding and 3D printing techniques have been extensively used to fabricate aerogels in desired combinations and shapes [54,55]. Using such techniques, it was possible to study the effect of each combination on the material performance and to control the inner pore structures precisely with high reproducibility [56]. Although these fabrication processes produce the desired shape, pore size and pore structure, due to the limited resources and limited funding, most of the studies at present are conducted by using freeze-drying or supercritical approaches as convenient techniques for lab-based and research evaluations.

### 2.3. Application of Nanocellulose-Based Aerogels

Nanocellulose-based aerogels have been utilized in a variety of applications depending on their type of cellulose, combined material(s) and modification to suit the desired application. The application of nanocellulose aerogels was further extended to biomedical and medical fields such as drug delivery and tissue engineering due to the biocompatibility and nontoxicity of nanocellulose [40]. Table 1 illustrates the different applications of nanocellulose-based aerogels for different fields.

## 3. Strategies for the Modification of Nanocellulose Aerogels to Suit Oil Absorption Application

**Figure 3:**
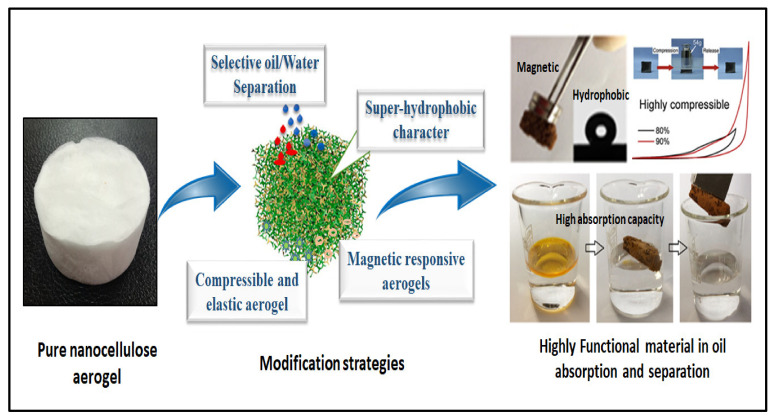
Illustration of commonly used modification strategies for nanocellulose aerogels to suit oil absorption and separation applications.

Aerogels based on nanocellulose possess great absorption capacity, giving this biopolymer promising potential in oil absorption applications. Despite the modification of the hydrophilicity of nanocellulose aerogels and the enhancement of their underwater stability, the removal of oil absorbents is yet another challenge [74]. Nanocellulose aerogels are known to have high porosity, ultralow density and high water absorption. Thus, modification of these materials is required for water treatment applications. Several approaches have been used to modify nanocellulose aerogels either by chemical treatments or the use of cross-linkers to enhance their hydrophobicity, induce their oil absorption selectivity and improve their shape retention and magnetic or pressure sensitivity (Figure 3).

### 3.1. Superhydrophobic Character

**Figure 4:**
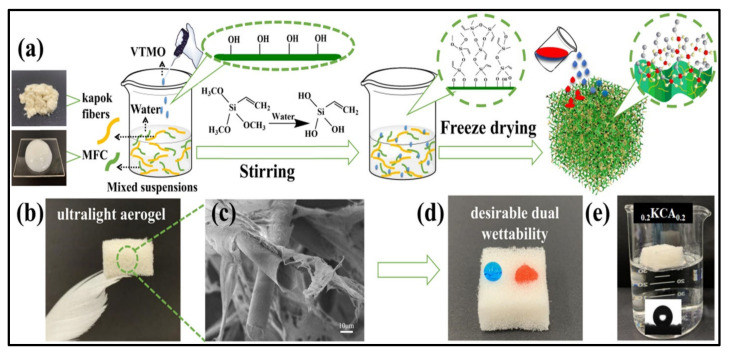
Illustration of hydrophobic cellulose-based aerogel properties: (**a**) schematic drawing of the fabrication approach using freeze-drying; (**b**) realistic image of prepared aerogel; (**c**) SEM image of the aerogel; (**d**) wettability characteristic of the aerogel; (**e**) water contact angle. Adapted from [79], with permission from Elsevier, 2022.

Biopolymers are known for their hydrophilic nature and high water absorbability, which can be serious issues in terms of water absorption applications. Several efforts have been made to develop hydrophobic biopolymer-based materials, including aerogels, for oil absorption applications. Although commercial materials such as polypropylene fiber mats have excellent hydrophobicity and good oil absorption capacities (around 15 g/g), they have the drawback of degradation, which is considered a remaining major environmental challenge [75]. Hydrophobic biopolymer-based aerogel, which can serve the same function with better performance, was developed to overcome this problem. Nanocellulose-based aerogels have been treated with octyl-trichlorosilane to obtain hydrophobic surfaces using vapor phase deposition [76]. The authors obtained superhydrophobic cellulose-based aerogel with absorption capacity comparable to that of commercial absorption materials. The combination of unique aerogel properties of high porosity and surface area and the use of abundant precursors such as plant wastes make the cellulose-based aerogels a highly promising candidate for these applications. In a recent study, nanocellulose aerogel was functionalized with a titanium dioxide layer by using atomic layer deposition [77]. Using the deposition approach produces highly effective absorbents, but this approach is complicated, expensive and requires the use of sophisticated equipment, which may increase the costs of production. To facilitate this and to minimize the costs, Feng et al. [78] developed a facile and cost-effective approach for the fabrication of a very stable superhydrophobic cellulose-based aerogel. The authors used paper waste as the source of cellulose and Kymene as a cross-linker. To modify the hydrophilicity of the cellulose aerogel, the authors coated the aerogel with methyltrimethoxysilane by using chemical vapor deposition, and they were able to achieve excellent oil (but not water) absorption capacities of more than 95 g/g by using only 0.25 wt.% of cellulose in the aerogel. Such recycled cellulose aerogels can be also developed from other wastes following the same approach, gaining the advantages of utilization of such wastes and the benefits of oil absorption. Zhang et al. [79] fabricated hydrophobic aerogel (140.1°) from kapok/microfibrillated cellulose possessing dual-scale hierarchically porous structure. In order to modify the hydrophilicity of cellulose, the authors dispersed the chopped kapok fiber into the aqueous solution of cellulose under magnetic stirring until uniform dispersion. Adding a few drops of vinyltrimethoxysilane and adjusting the pH to 4 was found to promote the hydrolyzation of the vinyltrimethoxysilane in addition to the formation of silanol groups that react with cellulose molecules and make them hydrophobic [79]. Figure 4 presents fabrication steps of hydrophobic cellulose aerogel with ultrahigh absorption ability for oils ranging from 104 to 190.1 g/g, which is comparable to and even higher than that of the conventional environmentally unfriendly aerogels.

Although numerous researchers have proposed reversing the hydrophilicity of a material intended for use in water treatment applications, others proposed using hydrophilic/oleophobic surface materials such as nanocellulose and chitosan for oil/water separation [80]. However, these materials lose their elaborate topological topography once they are placed underwater and thus cannot achieve hydrophilic/oleophobic properties [81]. Coating suitable-wettability biopolymers onto certain metal meshes or even textiles was found to be an effective approach in the preparation of hydrophilic/oleophobic materials [82]. Zhou et al. [83] developed a different approach for the fabrication of highly porous cellulose aerogel from softwood kraft pulp by using a facile silanization reaction for coating the aerogel with polysiloxane. The authors were able to produce superhydrophobic aerogel with a water contact angle as high as 151.8°. Polysiloxane enhanced the mechanical stability of the aerogel in addition to resulting in a huge oil absorption ability of up to 159 g/g. In different study, Dilamian and Noroozi [84] prepared cellulose-based aerogel with hydrophobicity of 151° by cross-linking rice straw cellulose with polyamideamine-epichlorohydrin followed by freeze-drying the mixture and then oven-heating it at 120 °C for 3 h to achieve covalent cross-linking and finally hydrophobic coating of the aerogel by using methyltrimethoxysilane under silanization reaction. The authors were able to achieve superhydrophobic aerogel with a water contact angle of 151°, dual-scale porous structure and excellent crude oil absorption performance (112 g/g). Such highly functional materials could be even more developed for better performance; the high porosity of aerogels promotes the high adsorption capacity, which is accelerated by the aerogel being modified to become superhydrophilic. This strategy is very important and has the advantage of making the aerogel reusable, as it can only absorb the oil but not the water. Although most of the previous works used chemical modifications to change the hydrophilicity of nanocellulose, tiny quantities are used and the material has the potential of being reusable several times. Using such modification techniques to further enhance the unique properties of nanocellulose aerogels could promote them to become the future replacements for commercial oil absorbents, as ecofriendly and biodegradable oil absorption materials.

### 3.2. Selective and Versatile Oil/Water Separation

**Figure 5:**
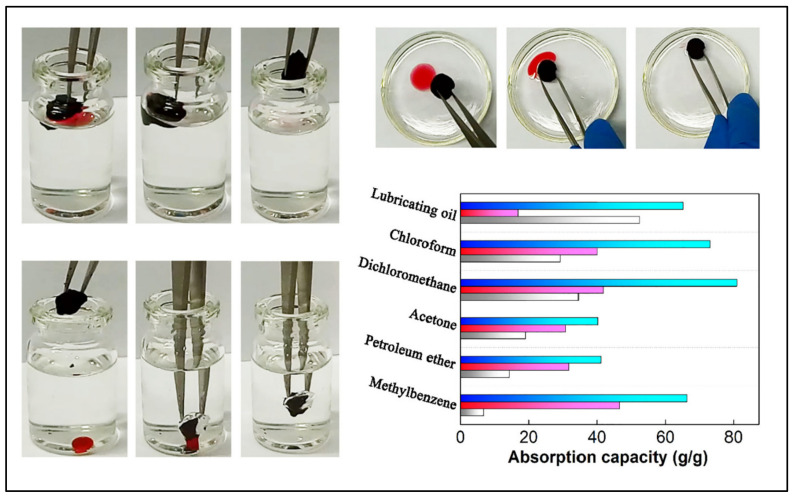
Nanocellulose/graphene aerogel in selective different oil absorption application, showing the high selectivity of the aerogel in oil absorption. Adapted from [90], with permission from Elsevier, 2022.

Oil selectivity is an important characteristic that increases the adsorption capacity of the aerogel and enhances its performance. Numerous materials have been incorporated with nanocellulose to increase its oil selectivity and thus enhance its hydrophobicity. Among them, graphene has attracted great attention as a hydrophobic surface material in the field of oil/water separation due to its excellent bonding with nanocellulose, good hydrophobicity, high specific surface area and unique chemical and mechanical stability [85]. Nguyen et al. [86] fabricated a facile approach to develop a superhydrophobic oil absorbent aerogel by coating melamine aerogel with graphene sheets using the dip-coating method. The aerogel exhibits great absorption capacity with excellent selectivity. The same principle was used in a different study to enhance the oil selectivity of nanocellulose, and surprisingly, the aerogel possessed even higher oil absorption capacity with excellent compressibility and recoverability [87]. In another investigation, Chatterjee et al. [88] developed a new approach to synthesize nanocellulose-based oil absorbent aerogel using polyethyleneimine and epoxy as chemical cross-linkers to enhance interaction and elastic recovery of the aerogel. The authors also dip-coated their aerogel with graphene nanosheets to maximize selective oil absorption, and they were able to achieve high selectivity with an absorption capacity reaching 25 to 58 g/g. The surface modification of the aerogel significantly improved the material selectivity in organic solvent absorption with the ability to rapidly retain shape after compression. Thus, mechanical squeezing can be applied for collecting the absorbed oil with over 100 squeezing–swelling cycles. In nanocellulose/graphene aerogels, polyethyleneimine is introduced as a cation part, which integrates with the nanocellulose chain and combines with the anion sheets of graphene [89]. Lu et al. [90] used this modification strategy and was able to fabricate nanostructured aerogel with a specific surface area of 441 m^2^/g. The aerogel showed superhydrophobicity with a water contact angle of 155.5°, which can be attributed to the increase in C–C and –NH groups in addition to the pyrolysis of hydrophilic groups. High selectivity toward the oil was observed in the aerogel; the authors reported extraordinary performance in terms of oil/water separation (Figure 5) [90].

Nanocellulose is known for its excellent absorption capacity, and such modification could be used to develop material in large size to address many environmental issues such as oil spills. A different technique was used by Li et al. [91], who used a simple approach to fabricate ecofriendly and highly functionalized cellulose aerogel. The authors coated the aerogel with copper nanoparticles to enhance its oil selectivity, and they were able to produce porous aerogel that can selectively collect oily contaminants in a few minutes with excellent absorption capacity and good recyclability. This production approach is facile, cost-effective and uses sustainable precursors, thus being is suitable for large-scale production without using any organic hydrophobic modification. Recent work has concluded that blending nanocellulose with graphene oxide leads to extensive hydrogen-bonding interactions between the two materials, which leads to a directionally aligned porous structure resulting from the typical porosity of nanocellulose aerogel and sheet type of graphene [92]. Such a blend gave the resulting aerogel prominent compression, elastic and high recoverability properties, resulting from the superoleophilicity of graphene, making the aerogel selectively collect oils with excellent absorption capacities [92]. In contrast, polycondensation of nanocellulose with three silane-coupling agents has been used to fabricate a hydrophilic–oleophobic aerogel with a robust compressive ability and selective water absorption from oil/water mixtures with high reusability [93]. Blending nanocellulose with a particular material and the fabrication approach determine the properties of the resulting aerogel in addition to its absorption capacity and/or hydrophobicity. Although the interaction between nanocellulose and one particular material may look the same in several works, different findings may be explained by the origin of nanocellulose as well as the type of additive and fabrication situations, which all have a great influence on the aerogel properties [56,94]. Although graphene may not be a green material, blending it with nanocellulose facilitates its degradation. Selective and versatile separation has the advantage of separating the two materials at the same time rather than absorbing one from the other, which makes the process faster, less expensive and suitable for large-scale applications. Combining aerogel with certain materials with specific ligands could give it the potential to be a selective absorbent for specific chemicals or drugs, which could open new doors for this functional material in pharmaceutical applications.

### 3.3. Magnetic Aerogels

**Figure 6:**
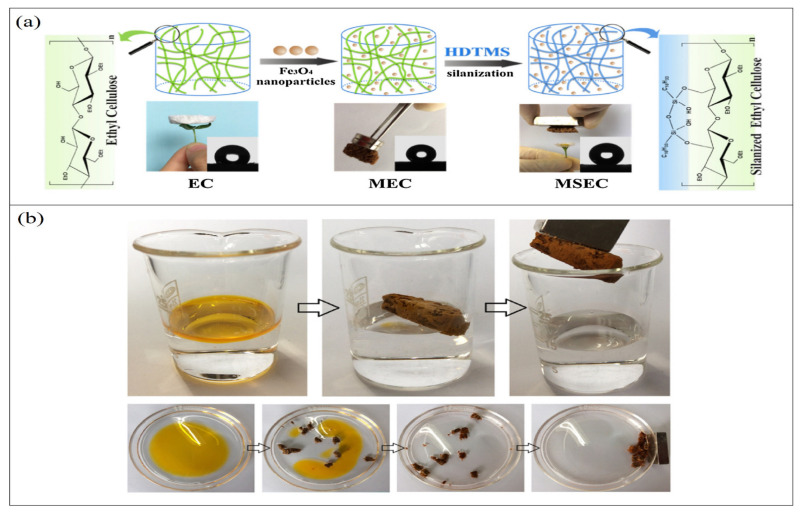
Illustration of modified ethyl cellulose magnetic aerogel: (**a**) schematic drawing of fabrication approach; (**b**) selective oil absorption and magnetic-responsive character. Adapted from [102], with permission from Elsevier, 2022.

The incorporation of iron oxide nanoparticles in cellulose-based aerogel to enhance the recovery ability of the aerogel by using a magnetic field has been gaining tremendous attention recently. The first attempt at this principle was made in 1973 by Turbeville, who used ferromagnetic sorbents for oil absorption and recovery [95]. Since then, a significant number of studies have been conducted using different iron compounds and different sorbent materials. Iron oxide nanoparticles have been reported to have excellent oil adsorption capacity besides their magnetic property [96]. However, due to the agglomeration and air oxidization characteristics, as highly chemically active compounds, these nanoparticles are not used in their naked form [97]. Nanocellulose aerogels have been proposed as a protecting shell on the surface of these nanoparticles, which can stabilize and maintain the function of iron oxide nanoparticles. A SiO_2_ shell should be used to modify Fe_3_O_4_ nanoparticles to enhance the compatibility of the two materials with the organosilanes and thus obtain uniform magnetic aerogels. Fe_3_O_4_ nanoparticles were found to increase the hydrophobicity and oleophilicity of the cellulose aerogel, in addition to enhancing its mechanical stability and increasing the roughness [98]. Chin et al. [99] used a facile approach to fabricate magnetic cellulose-based aerogel by incorporating magnetic Fe_3_O_4_ nanoparticles into the aerogel and then coating the surfaces with a thin layer of TiO_2_ to enhance the hydrophobicity of the aerogel. The authors used the sol–gel process for the coating process, and they were able to produce hydrophobic cellulose aerogel with the ability to easily be recovered from water by application of a magnetic field. Owing to the high porosity and the unique properties of cellulose, this aerogel was able to selectively absorb oil up to 28 times its own weight from a water/oil mixture in a short time. The use of iron oxide nanoparticles made the aerogel recovery quite easy upon the application of an external magnetic field. As an ecofriendly material, this type of biopolymeric aerogel has the potential of being the future solution for oil removal; it is an easy-to-prepare, unexpansive and selective oil absorbent with a great absorption capacity and efficiency. Dip-coating and adsorption approaches have been used to synthesize magnetic aerogels to transfer iron oxide nanoparticles into the aerogel texture [100]. Such an approach could also improve the hydrophobicity of nanocellulose aerogel in addition to its oil adsorption efficiency. However, it could lead to a slight loss of iron oxide nanoparticles during the oil adsorption process due to the weak bonding between nanocellulose and iron oxide, which could impairs its reusability [101]. In order to address this issue, a chemical cross-linker is used to enhance the bonding and immobilize the iron oxide nanoparticles within the aerogel system [99]. Such an aerogel was able to absorb up to approximately 28 times of the aerogel’s own weight within 10 min and possessed excellent recovery upon use of an external magnetic field. Lu et al. [102] developed for the first time an ecofriendly magnetic aerogel based on ethyl cellulose (Figure 6); the aerogel was superhydrophobic (θ water > 150°) in several kinds of solutions, with low density and high porosity, and was prepared by silanizing ethyl cellulose with hexadecyl-trimethoxysilane and then incorporating ferroferric oxide nanoparticles into the mixture. The authors were able to achieve 37–51 times absorption capacity for the prepared magnetic aerogel, which was found to be well maintained upon repeated use. This aerogel demonstrates superior recyclability compared with other magnetic aerogels.

Considering the poor selectivity of pure nanocellulose aerogels for oil, many researchers have introduced hydrophobic groups that possess low surface energy into the aerogel system to improve its oil adsorption capacity and introduced magnetic nanoparticles to improve the recovery of aerogel [103]. Lin et al. [104] treated nanocellulose aerogel with trimethyl-chlorosilane to improve its oil adsorption by using the low-temperature plasma technology and achieved an adsorption capacity of more than 34.5 g/g. The authors were able to enhance the adsorption capacity of the aerogel, but its removal and recovery were achieved in a similar study, which used the same approach and introduced Fe_3_O_4_ into the aerogel [105]. Rapid removal and easy reusability and separation of the adsorbent can be achieved by introducing magnetic property into nanocellulose aerogel under a permanent magnet. Regenerated cellulose was incorporated with manganese iron oxide (MnFe_2_O_4_) nanoparticles by in situ coprecipitation approach, using both sodium chlorite and sodium periodate as oxidants to achieve modified magnetic cellulose aerogels [106]. The addition of MnFe_2_O_4_ nanoparticles promotes the oil adsorption and enhances the mechanical stability of the aerogel. A different study used Fe_3_O_4_ nanoparticles in nanocellulose/inorganic silica fiber aerogel and reported that the addition of Fe_3_O_4_ nanoparticles significantly improved the aerogel surface area, water contact angle and compressive property, in addition to the adsorption capacity of the aerogel [107]. However, with the increase in Fe_3_O_4_ nanoparticles, the bulk density increased, and thus the adsorption capacity decreased. This strategy significantly facilitates the recovery of aerogel after the adsorption process, it can be used for large projects using large pieces of aerogel to address the marine oil spill issues. However, this strategy has the limitation of using iron nanoparticles, which could have adverse effects on ecosystems; currently, studies on the effects of iron oxides on human and environmental ecosystems are limited.

### 3.4. Compressibility and Elasticity (Shape Retention)

**Figure 7:**
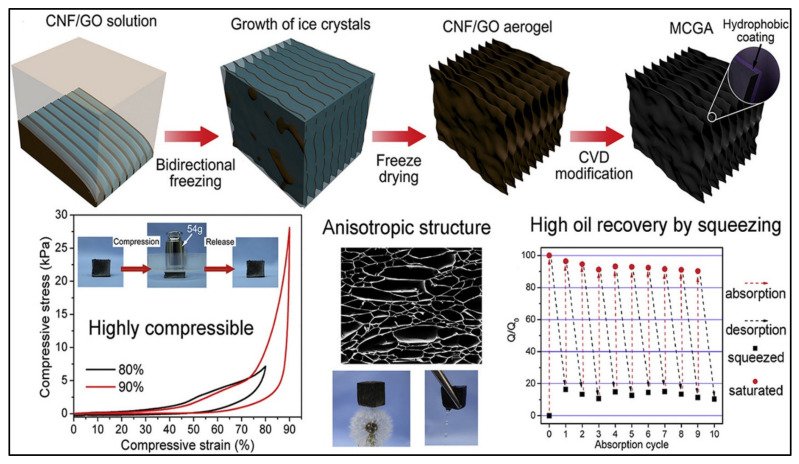
Illustration of the fabrication steps of compressible, anisotropic and elastic nanocellulose/graphene aerogel, showing its high oil recovery by squeezing. Adapted from [94], with permission from Elsevier, 2022.

Bioaerogels mostly possess low compressibility and elasticity due to their low density and high porosity. Preventing the permanent deformation and collapse of the polymeric network within the aerogel is the key to attaining aerogel with high compressibility and elasticity [108]. Nanocellulose aerogel has an anisotropic macroscopic structure; thus, permanent deformation and collapse of its structures occur upon the application of mechanical force. Designing the anisotropic porous structure of the aerogel was found to be a feasible solution for boosting the compressibility and elasticity of nanocellulose aerogels [109]. To prove this principle, Gao et al. [110] were able to fabricate a carbon–graphene monolith exhibiting an anisotropic lamellar structure with excellent compressibility and recoverability. The introduction of micro- and nanolattice structures into the material could even endow compressibility as well as elasticity to rigid metals and fragile ceramics [111]. Recent investigations showed that cross-linking nanocellulose with graphene oxide increases the elasticity of the resulting aerogel and enhances its compression [112]. Mi et al. [94] modified nanocellulose to produce 3D highly compressible, anisotropic and elastic aerogel using a bidirectional freeze-drying approach. The authors incorporated graphene aerogels with nanocellulose to produce hybrid aerogel and then modified it by using the DDTS approach, which includes using saline with a long carbon chain, and they were able to produce highly compressible superhydrophobic aerogel (Figure 7). The outstanding compression and recoverability properties of the aerogel came as a result of mixing the properties of flexible cellulose and stiff graphene. Upon the compression of aerogel to 60% and 90%, strain the authors were able to recover 99.8% and 96.3%, respectively. Owing to the ultralight weight of the aerogel and high surface area, its absorption capacity was extremely higher than that of conventional aerogel and ranged from 80 to 197 times its weight. Such compressible aerogel also possesses efficient recovery of oil upon simply squeezing the aerogel. The authors claim that the high compressibility of their aerogel and its high oil recovery ability have never been obtained by any other cellulose-based aerogel. This strategy enhances the mechanical stability of the material, making it remain intact and avoiding any possible leaching that may occur in the environment. Future studies should use more green materials to enhance the compressibility of aerogels to avoid any possible long- or short-term adverse effects on humans and/or environment after their use.

## 4. Biopolymer Aerogel Composites for Oil Absorption and Separation Applications

**Figure 8:**
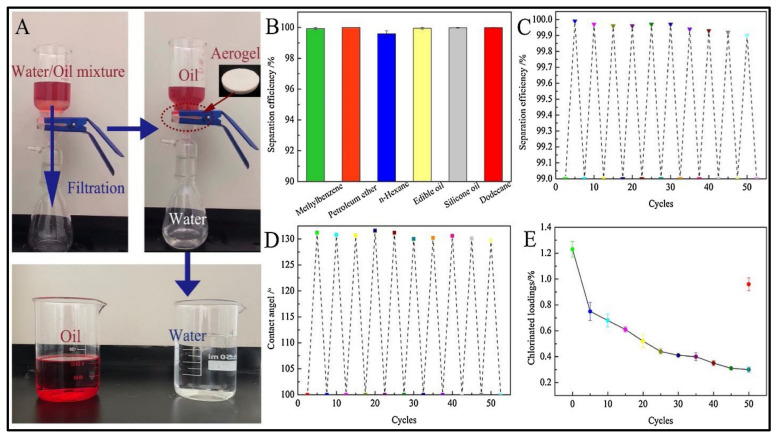
Illustration of the great performance of nanocellulose/chitosan aerogel in oil/water separation, showing the separation of the two materials in high purity; (**A**) oil/water separation experiment, (**B**,**C**) separation efficiency values, (**D**) water contact angle, and (**E**) chlorinated loadings. Adapted from [113], with permission from Elsevier, 2022.

The use of biodegradable materials to address oil spill issues has attracted significant attention in the past few years. Owing to the unique properties of modified nanocellulose-based aerogels, they could serve as the future materials for all oil absorption and separation purposes. In recent work, Korhonen et al. [77] fabricated a modified nanocellulose aerogel by using the atomic layer deposition technique to coat the aerogel with a TiO_2_ layer and used it for oil removal. The authors were able to achieve good oil absorption capacity with high selectivity, but the approach requires the use of sophisticated equipment that raises its costs compared to conventional removal and absorption approaches. To solve this problem and reduce the production costs, Chin et al. [99] developed a facile approach to synthesize superhydrophobic and magnetic nanocellulose aerogel by incorporating magnetic nanoparticles within the nanocellulose and coating the surfaces with a thin TiO_2_ layer. The resulting aerogel was able to rapidly absorb the oil with an absorption capacity of 28 times the aerogel weight in a few minutes. This unique aerogel was also magnetically sensitive, and the authors were able to recover it without reducing its absorption capacity. In a recent study, Zhang et al. [113] developed a novel aerogel system for oily wastewater treatment, using nanocrystalline cellulose and chitosan as the major precursor materials. The authors used a quaternized N-halamine siloxane monomer to modify the hydrophilicity of the aerogel by hydrolyzing it into polymer form in the nanocellulose/chitosan solution. Owing to the hydrophilic nature of nanocellulose and the porous structure of aerogel that endow the prepared aerogel with underwater oleophobic character, the authors were able to achieve high separation efficiency for several types of oil/water solutions with separation efficiency close to 100% (Figure 8). Furthermore, the reusability of such aerogel did not affect the separation performance; thus, it could be a promising material for different oil/water separation applications.

Numerous publications apply multiple modifications to the same aerogel to enhance its absorption performance in terms of superhydrophobicity, compressibility and ease of reusability. Compared with the conventional commercial materials such as polypropylene and its derivatives that possess an absorption capacity of 8.1–24.6 g/g and a water contact angle of about 102.1° [114], most of the fabricated cellulose aerogel possess superior performance. Table 2 presents a summary of the most recent publications on nanocellulose-based aerogels in oil absorption applications compared with polypropylene.

## 5. Potential and Future Perspective of Nanocellulose-Based Aerogels

In recent years, several novel adsorbent and separation materials have appeared, such as graphene sponges and aerogels, carbon nanotube frameworks, carbon fiber aerogels and biopolymer-based aerogels [124,125]. Although these materials possess excellent oil/water adsorption and/or separation performance, most of them are nonbiodegradable, which could raise another issue for the environment. Among them, biopolymer-based aerogels have been modified to become highly competitive to inorganic adsorbents, with minimal disadvantages. In comparison, the modified nanocellulose-based aerogels proved to have a superhigh oil-absorbing capacity and can also be reused for several cycles without significant reduction in the adsorption capacity. Although nanocellulose-based aerogels for oil absorption and separation have not yet entered commercialization and are still limited to the research and laboratory sectors, we believe that the following years will witness great advances in the commercialization of these materials.

Great advances have been made in the past few years in terms of fabrication techniques; the traditional approaches that use excessive amounts of organic solvents are no longer applicable, which is significantly reducing the production costs [20]. The use of freeze-drying may consume time and energy, but the following years will witness modified printing techniques that will enhance the fabrication of such functional materials that can maintain the function and achieve the possibility of large-scale production and commercialization of nanocellulose-based aerogels. The further functionalization and multifunctionality of nanocellulose aerogels in different water treatment applications have encountered several challenges, including designable structures, undesirable dispersion of fillers within the aerogel, modification of surface functional groups and the possibility of transition from laboratory and research into industrialization. Future studies should consider the mentioned challenges and comprehensively consider subjective initiation to develop the applications and determine the future role of nanocellulose-based aerogels in future life. However, many functional fillers have been already incorporated with nanocellulose aerogels to endow structurally anisotropic functionalities such as magnetism and electricity, thereby easing their function in water treatment and broadening their application fields. Such studies are still in their initial stage and require further laboratory and economical evaluation before they reach industrialization.

## 6. Conclusions

Nanocellulose aerogels are a new category of high-efficiency adsorbents for treating oil spills and water pollution. They not only present typical characteristics of high porosity, large specific surface area and light weight but also incorporate excellent inherent properties such as high availability, low cost, easy scale-up, nontoxicity and sustainability. Although most of the strategies for modifying nanocellulose aerogels involve physical blending or chemical modification, nanocellulose is still the major part of the material. Such modifications can endow nanocellulose aerogels with additional special features, including superhydrophobicity, oil absorption selectivity, improved shape retention and magnetic or pressure sensitivity. Therefore, apart from oil/water separation, nanocellulose aerogels have great application prospects in other water treatment applications such as the adsorption of pesticides, herbicides, pharmaceuticals and heavy metals.

## Figures and Tables

**Table 1:** The role of nanocellulose-based aerogels in different applications.

Field of Application	Application	Type of Aerogel	Remark	Ref.
Environmental	Absorption of oils and organic solvents	Cellulose/chitosan aerogel	Highly hydrophobic aerogel from cross-linking oxidized cellulose with chitosan and cold plasma modification	[56]
	Removal of chemicals	Cellulose-based aerogel	Polyaniline used as interface layers; the aerogel had high absorption capacity (409.55 mg/g) for tetracycline	[57]
	Removal of heavy metals	Robust cellulose aerogel	Polyethylenimine used as cross-linker; the aerogel showed high removal capacity (163.4 mg/g), fast adsorption rate and high shape recovery	[58]
	Water treatment	Cellulose nanocrystals/poly(methyl vinyl ether-co-maleic acid)/poly(ethylene glycol) aerogel	The chemically cross-linking aerogel was able to absorb all the cationic dyes from water (116.2 mg/g)	[59]
	Air purification	Robust micro-honeycomb-like nanofibrous aerogels	The aerogel showed extraordinary filtration performance even for 0.3 μm sized particles	[60]
Industrial	Enzyme immobilization	Bacterial cellulose/poly(glycidyl methacrylate) aerogel	The bacterial cellulose was modified with PGMA through atom transfer radical polymerization approach for catalase enzyme immobilization	[61]
	Protein separation	Dendrimer-assisted boronate affinity cellulose	Rapid adsorption rate was achieved with outstanding adsorption capacity for proteins (537.4 mg/g)	[62]
	Thermal insulation	Gelatin/hydroxyethyl cellulose–SiO_2_	Hydrogen bonding and chemical cross-linking led the thermal conductivity of aerogel being lowered to 0.035 W/m K	[63]
	Packaging	Arundo donax cellulose aerogel	The superabsorbent bioactive aerogel reduced oxidation processes in red meat, leading to the extension of its shelf life	[64]
Biomedical	Drug delivery	Bamboo shoot cellulose/sodium alginate aerogels	Curcumin initially encapsulated in the aerogel, which then released in a sustained manner	[65]
	Biosensing	Triarylmethane-loaded cellulose acetate aerogel	Urease enzyme was used as a catalytic agent in the aerogel for colorimetric detection of urea	[66]
	Wound healing	Cellulose/konjac glucomannan	The biocompatible aerogel enabled faster wound recovery by enhancing cell proliferation	[67]
	Tissue scaffolding	Cellulose nanofiber–gelatin aerogel	Epichlorohydrin was used as a cross-linker; the aerogel showed adequate cytocompatibility and cell viability	[68]
	Antimicrobial	Nanocellulose aerogel loaded with thymol	The aerogel possessed a high effect against Gram-positive and -negative bacteria in addition to yeasts	[69]
Others	Electric conductivity	Bacterial cellulose/graphene oxide aerogels	The addition of dimethyl sulfoxide enhanced the electric conductivity of the aerogel	[70]
	Catalyst	Gold nanoparticles supported on cellulose aerogel	Cellulose aerogel was loaded with gold nanoparticles and possessed high yield and selectivity for styrene epoxidation	[71]
	Supercapacitor	Carbonized cellulose nanofibril aerogel	The aerogel had excellent electrochemical stability even after 5000 cycles and still kept 89.43% of its specific capacitance	[72]
	Flame retardancy	Bulk Al-doped carboxymethyl cellulose aerogels	The aerogel had better weight-bearing capacity than conventional silica aerogel and could be rated V-0 in terms of UL-94 testing for flame retardancy	[73]

**Table 2:** Literature summary of nanocellulose-based aerogels for oil absorption applications.

Type of Aerogel	Preparation Method	Absorption Capacity (g/g)	Water Contact Angle	Outcomes	Ref.
Polypropylene-based material (reference)	Commercial material	8.1–24.6	102.1°	The hollow fiber had higher absorption rate than solid fibers	[114]
Budget cotton-based aerogel	CO_2_ supercritical drying and vapor deposition	16.0	153.0°	Simple, rapid and effective superhydrophobic material for oil absorption	[115]
Bacterial cellulose and fumed silica aerogel	Freeze-drying of fumed silica and infiltrated bacterial cellulose	28	142.0°	Used 10 times for oil recovery without any reduction in the uptake capacity	[116]
Modified waste cellulose fibers	Freeze-drying	142.9	159.0°	Super oil absorption capacity for at least 30 cycles	[117]
Anisotropic graphene oxide/polyvinyl alcohol/CNF carbon aerogel	Freeze-drying	155–288	140.0°	Outstanding compressibility and recyclability with extremely large oil absorption capacity	[118]
Nanocellulose/nanochitosan/reduced graphene aerogel	Hydrothermal and freeze-drying technique	120–176	115.2°	Oil/water pump apparatus containing the aerogel continuously removed/collected the oil from wastewater	[119]
Wool waste fiber aerogel	Direct freeze-drying	136.2	138.0°	9.1–15.3 times better than commercial oil absorption materials	[120]
Rice straw nanocellulose aerogel	Freeze-drying	170	151.0°	Superlative adsorbent for remediation of polluted water	[84]
Bagasse-based aerogels	Freeze-drying and thermo-crosslinking	31.65	148.0°	High oil absorption capacity and low thermal conductivity	[121]
Collagen and dialdehyde carboxymethylcellulose	Freeze-drying and surface coating	20.4–57.2	144.4°	Excellent reusability and recyclability for oily liquids	[122]
Kapok/microfibrillated cellulose aerogels	Vacuum freeze-drying and surface modification	104–190.1	140.1°	Rapid, selective and ultrahigh absorption and recycling ability	[79]
Raw cotton fiber macroporous cellulose aerogel	Sol–gel and freeze-drying techniques	19.8–41.5	154.0°	The aerogel possessed superb oil retention capability	[123]
Nanocellulose/silica fiber/Fe3O4 aerogel	Direct freeze-drying and surface modification	34.2–58.3	150.0°	Superior stability in wide pH range with multiple uses	[107]
Graphene/cellulose/silica aerogel	Hydrothermal and subsequent freeze-drying	39–68	157.0°	Extraordinary absorption efficiency for various oils	[87]

## Data Availability

Not applicable.
